# Role of Endoplasmic Reticulum and Oxidative Stress Parameters in the Pathophysiology of Disease-Related Malnutrition in Leukocytes of an Outpatient Population

**DOI:** 10.3390/nu11081838

**Published:** 2019-08-08

**Authors:** Celia Bañuls, Aranzazu M. de Marañón, Iciar Castro-Vega, Sandra López-Doménech, Irene Escribano-López, Christian Salom, Silvia Veses, Antonio Hernández-Mijares

**Affiliations:** 1 Service of Endocrinology, University Hospital Doctor Peset, Foundation for the Promotion of Health and Biomedical Research in the Valencian Region (FISABIO), 46017 Valencia, Spain; 2 Institute of Health Research INCLIVA, University of Valencia, 46017 Valencia, Spain; 3 Department of Medicine, University of Valencia, 46017 Valencia, Spain

**Keywords:** disease-related malnutrition, inflammation, mitochondrial function, endoplasmic reticulum stress, outpatient population

## Abstract

Cellular pathways such as inflammation or oxidative stress are the cause and triggers of disease-related malnutrition (DRM), but the influence of these markers on endoplasmic reticulum (ER) stress is unknown. The objective of this study was to analyze the relationship between mitochondrial function and ER stress parameters in a DRM population. The study population was composed of 82 outpatient subjects, of whom 45 were diagnosed with DRM and 37 were confirmed to be normonourished according to the American Society for Parenteral and Enteral Nutrition ASPEN criteria. We evaluated anthropometrical and biochemical parameters, pro-inflammatory cytokines in serum. Oxidative and ER stress markers were analyzed in leukocytes. DRM patients showed significant reductions in albumin and transferrin concerning the normonourished group, and also displayed higher levels of hsCRP, IL6, and TNFα, and the soluble adhesion molecules VCAM-1 and ICAM-1. Besides, oxygen consumption and mitochondrial membrane potential were reduced and Radical Oxygen Species ROS production was enhanced in DRM patients. In the case of ER markers, protein and mRNA expression revealed that CHOP, ATF6, and the P-eIF2α signal were enhanced in malnourished patients compared to control subjects. Correlation studies supported a relationship between weight loss and increased inflammation, mitochondrial dysfunction, and compromised function of the ER. Our results demonstrate that ER stress signaling pathways are influenced by inflammation and mitochondrial function in the leukocytes of a DRM population.

## 1. Introduction

The interaction between the nutritional status of a subject and disease can result in disease-related malnutrition (DRM). This is a multifactorial condition caused by a deficit of nutrients, and is triggered by poor nutrient utilization, increased nutrient loss, and heightened nutrition requirements [1,2,3].

Decrease of nutrient intake is a common physiopathological mechanism, which in itself entails an increased risk of malnutrition, in addition to an inflammatory response [3,4]. Besides the abovementioned consequences, the diminished intake of nutrients impacts negatively on the modulation of the immune system, thus favoring the overproduction of inflammatory mediators [4].

In this context of chronic inflammation, a wide range of diseases associated with DRM (cancer, respiratory, neurodegenerative, and digestive diseases), are also related to the overproduction of reactive oxygen species (ROS). Additionally, individuals are more prone to present oxidative stress if there is a lack of antioxidants together with malnutrition [5]. This situation of increased cellular stress can undermine the adaptive capacity of the endoplasmic reticulum (ER). ER plays a pivotal role in maintaining cellular and metabolic homeostasis [6,7]. Upon ER stress, unfolded protein response (UPR) can be activated, intersecting with many stress signaling pathways [8]. Several mediators and transcription factors are activated during UPR, including 78-KDa glucose-regulated protein (GRP78), activating transcription factor 6 (ATF6) and phosphorylated eukaryotic translation initiation factor 2 subunit 1 alpha (P-eIF2α), thus increasing the ER ability of degrading unfolded proteins [9]. Growing evidence suggests that this pathway, at some point, merges with inflammatory pathways and together they interfere in the metabolic functions. In fact, in animal models, it has been shown that mitochondrial dysfunction produces ER stress and deregulated lipid metabolism as a result of impaired autophagy [10].

To date, the cellular and molecular mechanisms underlying DRM are unclear. Our unpublished data showed that DRM is associated with mitochondrial dysfunction and an inflammatory state; however, it is still unknown how these processes can influence the DRM in leukocytes—a cell type determinant for the inflammatory response—is an issue that requires further research. Therefore, the objective of this preliminary study was to evaluate the connection between mitochondrial function and ER stress parameters in leukocytes from a DRM outpatient population and to compare it with a group of normonourished subjects.

## 2. Material and Methods

### 2.1. Subjects

Forty-five DRM patients and 37 normonourished (NN) subjects were recruited at the Endocrinology and Nutrition Service of the University Hospital Dr. Peset and adjusted by age and sex. Exclusion criteria were age under 18, pregnancy or lactation, oedema, dehydration, and severe renal or hepatic disease. All DRM and NN patients suffered some type of disease, which are detailed in Table 1.

The study was conducted according to the ethical principles stated in the Declaration of Helsinki. All procedures were approved by the Ethics Committee of the hospital. Written informed consent was obtained from all subjects.

### 2.2. Assessment of Nutritional Status

Nutritional status was determined following the American Society for Parenteral and Enteral Nutrition ASPEN criteria [11]. Medical and dietary-nutritional history, pharmacological treatment, physical examination, anthropometric measurements, and laboratory data were recorded for evaluation. Body weight and height were determined using an electronic scale and a stadiometer, respectively. In patients unable to stand, the size was estimated according to the length of the forearm. Body mass index (BMI) was calculated using the BMI formula (Equation (1)) as following:

BMI = weight (kg)/(height^2^ (m^2^)) (1)

Other parameters, including triceps skinfold thickness (TST) and mid-upper arm circumference (MUAC), were measured on the non-dominant arm. TST was assessed employing a skinfold calliper (Holtain LTD, Crymych, UK) and MUAC was determined at the middle of the arm with a non-elastic tape measure. Arm muscle perimeter (AMP) was determined using the formula (Equation (2)). Calf circumference (cm) was measured with a non-elastic tape measure.

AMP (cm) = MUAC (cm) − (TST (mm) × 0.314)(2)

### 2.3. Blood Sampling

Venous blood was drawn from patients after 12 h fasting and was centrifuged (1500 g, 10 min, 4 °C) for serum collection. Biochemical parameters were assessed in fresh samples and the remaining serum was stored in aliquots at −80 °C for later assays.

### 2.4. Biochemical Determinations

Biochemical determinations were carried out by our hospital’s clinical analysis service. Albumin concentration was determined using the Bromocresol green method (Abbott Laboratories, Chicago, IL 60064, USA), with a CV of 3.3% and a sensitivity of 0.3 g/dL. A nephelometric method employing a Beckman LX-20 autoanalyzer (Beckman Coulter, Brea, CA, USA) was used to determine prealbumin, transferrin, and high-sensitivity C-reactive protein (hsCRP), with a CV of <5%.

### 2.5. Soluble Cytokines and Adhesion Molecule Assay

Intercellular adhesion molecule 1 (ICAM-1), vascular cell adhesion molecule-1 (VCAM-1), the interleukin 6 (IL6) and tumor necrosis factor α (TNFα) were analyzed in serum samples with a Luminex 200 flow analyzer system (Millipore, Austin, TX, USA) samples. We followed the manufacturer’s instructions for each kit. The intra-serial CV was <5.0%, and the interserial CV was <15.0%, for all determinations.

### 2.6. Leukocyte Isolation

Approximately 25 ml of the blood obtained was incubated for 45 min with dextran (3% in saline solution). After incubation, the supernatant was collected, laid over 15 ml Ficoll-Hypaque and centrifuged at 250 g for 25 min. Subsequently, erythrocytes were lysed with Erythrocyte Lysis Buffer for 5 min, centrifuged at 1200 rpm for 5 min, and washed with PBS. The pellet was then resuspended in HBSS, centrifuged at maximum speed for 5 min, and stored at −80 °C for subsequent determinations.

### 2.7. Static Cytometry

Total ROS content, cytosolic calcium content, mitochondrial ROS content, and mitochondrial membrane potential were measured by employing 2′,7′-dichlorodihydrofluorescein diacetate (DCFH-DA) 5 μM, Fluo-4 1 μM, MitoSox 1 μM and Tetramethylrhodamine (TMRM) 5 μM, respectively. Leukocytes were plated in 48-well plates in HBSS and left until cells adhered to the surface. Subsequently, fluorophores were added and incubated for 30 min at 37 °C under gentle shaking, after which cells were washed. Images were recorded with an IX81 Olympus fluorescence microscope (Olympus, Hamburg, Germany), capturing 12 images per well. Quantification of images was performed with a coupled static cytometry software ‘ScanR’ version 2.03.2 (Olympus). All the fluorescent probes were purchased from Invitrogen (Life Technologies, Barcelona, Spain).

### 2.8. Mitochondrial Oxygen Consumption Measurements

The mitochondrial oxygen consumption rate was analyzed on a total of 5 × 10^6^ leukocytes per sample. The leukocytes were resuspended in HBSS and put in an oxygen-tight chamber coupled to a Clark-Type O_2_ electrode (Rank Brothers, Bottisham, UK). To confirm that mitochondria were the source of oxygen consumption, sodium cyanide was employed (10^−3^ mol/l). Duo 18 software (World Precision Instruments, Stevenage, UK) was used for data acquisition. Cell viability was confirmed with Trypan Blue and Scepter Cell Counter (MilliporeSigma, Burlington, MA, USA).

### 2.9. Protein Extraction and Quantification

Leukocytes were resuspended in lysis buffer (20 mM HEPES pH 7.5, 400 mM NaCl, 20% Glycerol, 0.1 mM EDTA, 10 μM Na_2_MoO_4_, 0.5% NP-40) containing protease inhibitors and Dithiothreitol 1 mM and then laid on ice for 15 min. Next, the tubes were vortexed for 30 s and centrifuged at 15,000 g at 4 °C, The protein supernatant fractions were collected and quantified using the BCA protein assay kit (Thermo Fisher Scientific, Rockford, IL, USA).

### 2.10. Western Blot

A total of 25 μg of the extracted protein was resolved by SDS-PAGE. In brief, samples were prepared with Laemmli Buffer and molecular water to equal volumes. The samples were boiled at 95 °C and then centrifuged for collecting the whole sample. Samples were separated on 10–13% acrylamide gel at 120 mV for approximately 1 h and transferred to nitrocellulose membranes at 400 mA, at 4 °C, for 1 h using a wet transference method. The membrane was then stained with Ponceau Red and washed twice with TBST. The primary antibodies (anti-GRP78 rabbit polyclonal antibody (Abcam, Cambridge, UK), anti-ATF6 mouse monoclonal antibody (which detects the cleaved/active form of ATF6; Thermo Scientific, Rockford, IL, USA), anti-CHOP mouse monoclonal antibody (Thermo Scientific, Rockford, IL, USA), anti-eIF2α-pS52 (Life Technologies, Barcelona, Spain), and anti-actin rabbit polyclonal antibody (Sigma Aldrich, St. Louis, MO, USA) were incubated overnight at 4 °C under gentle shaking. Membranes were then washed twice in TBST and blotted with the secondary antibody for 1 h at RT. The membranes were then incubated with Supersignal West Femto (Thermo Scientific, Rockford, IL, USA). Protein signal was measured using a Fusion FX5 Acquisition System (Vilbert Lourmat, Marne La Vallée, France). Subsequent quantification of the protein by densitometry was performed using Bio1D software (VilbertLourmat, Marne La Vallée, France). Data was referred to the actin signal and an internal control used in all the experiments.

### 2.11. RT-PCR

Patients leukocyte samples were selected and RNA extracted with the GeneAllR Ribospin^TM^ total RNA extraction kit (GeneAll Biotechnology, Seoul, Korea). The RNA quantity and purity were analyzed using Nanodrop 2000c (Thermo Scientific, Rockford, IL, USA). First-strand cDNA was synthesized by employing 1μg of the isolated RNA using a RevertAid first-strand cDNA synthesis kit (Thermo Scientific, Rockford, IL, USA). The gene expression was assessed with 1/10 dilutions of cDNA using KAPA SYBR FAST Universal Master Mix (KAPA Biosystems, Boston, MA, USA) in a 7500 Fast RT-qPCR system (Life Technologies, Carlsbad, CA, USA). The primer sequences are the following: *CHOP*: Fwd: AGAACCAGGAAACGGAAACAGA and RW: TCTCCTTCATGCGCTGCTTT; GRP78: Fwd: AAGAACCAGCTCACCTCCAACCC and RW: TTCAACCACCTTGAACGGCAA. Results were quantified using the ∆∆CT method, relativizing the data to the internal control and the GAPDH signal. Analysis was performed with Expression Suite software (Life Technologies, Carlsbad, CA, USA).

### 2.12. Statistical Analysis

SPSS software was used for data analysis. The values in the tables are mean ± standard deviation (SD) when data are parametric or median, 25th and 75th percentiles when data are non-parametric. Data in the bar graphs are mean ± standard error (SE). The parametric data were compared with a Student’s *t*-test, and non-parametric data were compared with a Mann–Whitney U test. Correlations were examined by Spearman’s correlation coefficient. Significant differences were considered when *p* < 0.05.

## 3. Results

### 3.1. Anthropometric, Metabolic, and Biochemical Parameters

A total of 82 outpatient subjects were recruited, of whom 45 were DRM patients and 37 were normonourished subjects according to ASPEN criteria. Both populations were homogeneous regarding gender or age but significant differences were found in terms of weight (*p* = 0.021), weight loss (*p* < 0.001), and BMI (*p* < 0.001), as shown in Table 2 Also, the malnourished group displayed lower tricipital skin thickness (*p* < 0.001), mid-upper arm circumference (*p* < 0.001), arm muscular circumference (*p* < 0.001), and calf circumference (*p* < 0.001) comparing with the normonourished group (Table 2).

When metabolic parameters were analyzed, significant reductions were found in albumin (*p* < 0.001) and transferrin (*p* = 0.031) in the DRM vs. NN group. As expected, the DRM group showed higher levels of hsCRP (*p* = 0.038) than NN subjects as seen in Table 2. These parameters are not strictly diagnostic for the nutritional state, even more in our population in which caloric malnutrition is more present (80.2% of the DRM subjects) but it strengthens the low nutritional state in which these subjects are. Also, hsCRP presents significant differences attributable to the nutritional state but not at a diagnostic level.

### 3.2. Inflammatory Status

**Figure 1:**
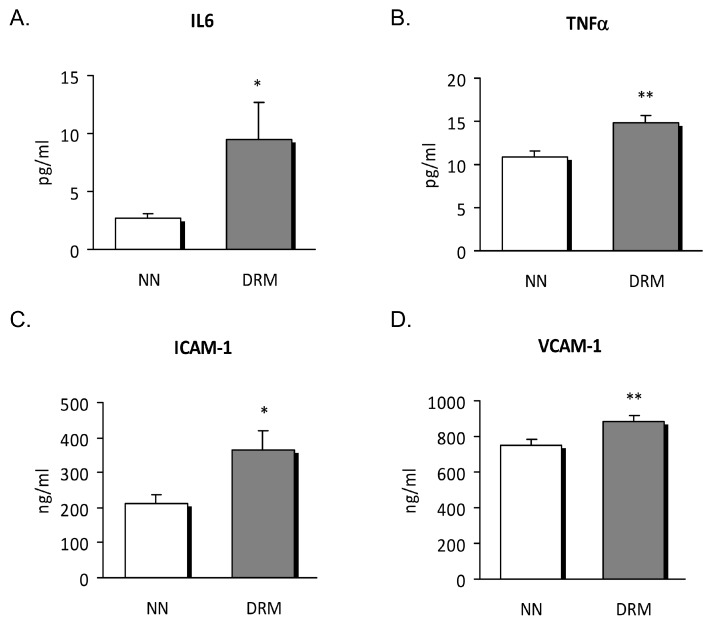
Serum levels of cytokines and soluble adhesion molecules from NN and DRM subjects. (**A**) IL6 levels measured as pg/ml. (**B**) TNFα levels measured as pg/ml. (**C**) ICAM-1 levels measured as ng/mL. (**D**) VCAM-1 levels measured as ng/ml. * *p* < 0.05 and ** *p* < 0.01 vs. NN group using an unpaired *t*-test. NN: Normonutrition; DRM: Disease-related malnutrition.

To corroborate the inflammatory status of the subjects, we also assessed inflammatory cytokines and soluble adhesion molecules in serum samples from normonourished and DRM patients. This analysis revealed higher levels of the pro-inflammatory cytokines IL6 and TNFα, and soluble ICAM-1 and VCAM-1 in our DRM patients (Figure 1). As a whole, these data highlight the pro-inflammatory status of DRM patients with respect to normonourished subjects.

### 3.3. Mitochondrial Function

**Figure 2:**
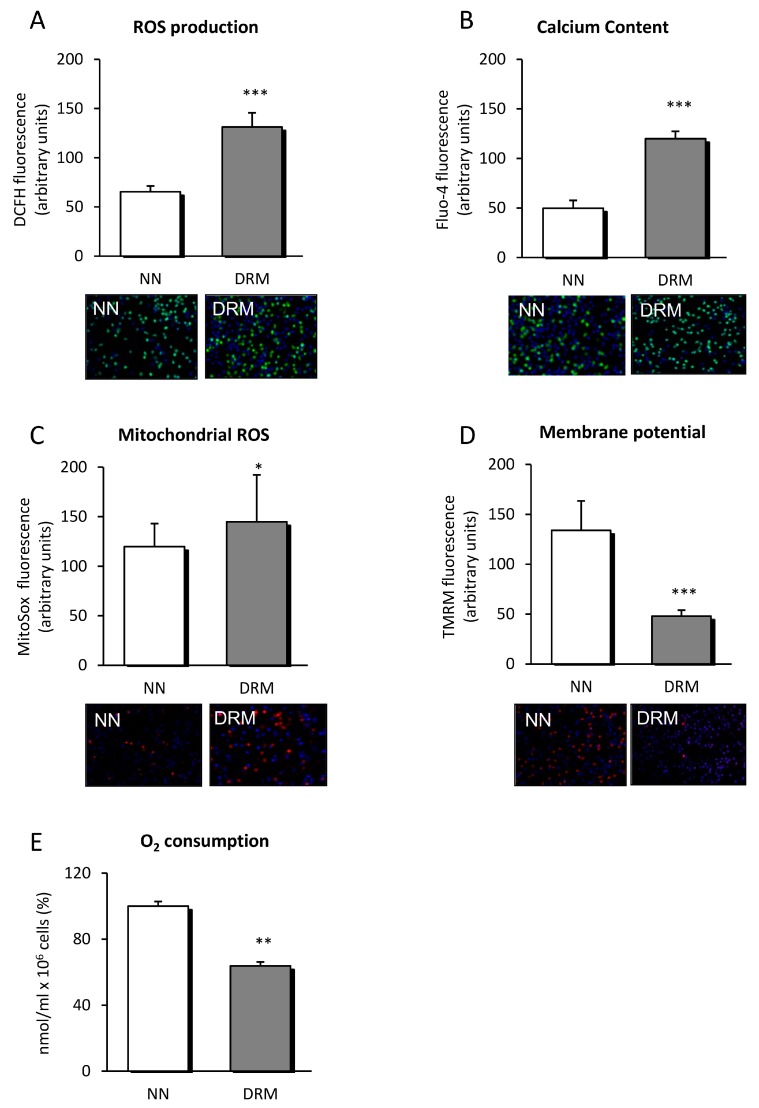
ROS production, calcium content and parameters of mitochondrial function measured in leukocytes from NN and DRM patients. (**A**) ROS production of leukocytes from NN and DRM subjects determined by measuring DCFH fluorescence in arbitrary units. Representative fluorescence images of both groups are displayed below the graph. (**B**) Calcium content of leukocytes from NN and DRM subjects determined by measuring Fluo-4 fluorescence in arbitrary units. Representative fluorescence images of both groups are displayed below the graph. (**C**) Mitochondrial ROS content of leukocytes from NN and DRM subjects, measured by TMRM fluorescence, in arbitrary units. Representative fluorescence images of both groups are displayed below the graph. (**D**) Mitochondrial membrane potential of leukocytes of NN and DRM subjects measured by TMRM fluorescence in arbitrary units. Representative fluorescence images of both groups are displayed below the graph. (**E**) Oxygen consumption of leukocytes from NN and DRM subjects with an oximeter. * p < 0.05, ** *p* < 0.01 and *** *p* < 0.001 vs. NN group using an unpaired *t*-test. NN: Normonutrition; DRM: Disease-related malnutrition; ROS: Radical oxygen species; DCFH: 2′,7′-Dichlorofluorescein diacetate.

As ER stress influences mitochondrial function, we analyzed certain mitochondrial parameters. Amounts of total and mitochondrial ROS were higher in DRM patients (Figure 2A,C), and oxygen consumption was reduced (Figure 2E), suggesting that mitochondrial function was compromised. This possibility was reinforced by the mitochondrial membrane potential data, which showed lower values in DRM patients (Figure 2D).

Additionally, calcium content was significantly enhanced in DRM patients suggesting calcium metabolism being dysregulated, maybe by ER function also being undermined (Figure 2B).

### 3.4. Endoplasmic Reticulum Stress Analysis

**Figure 3:**
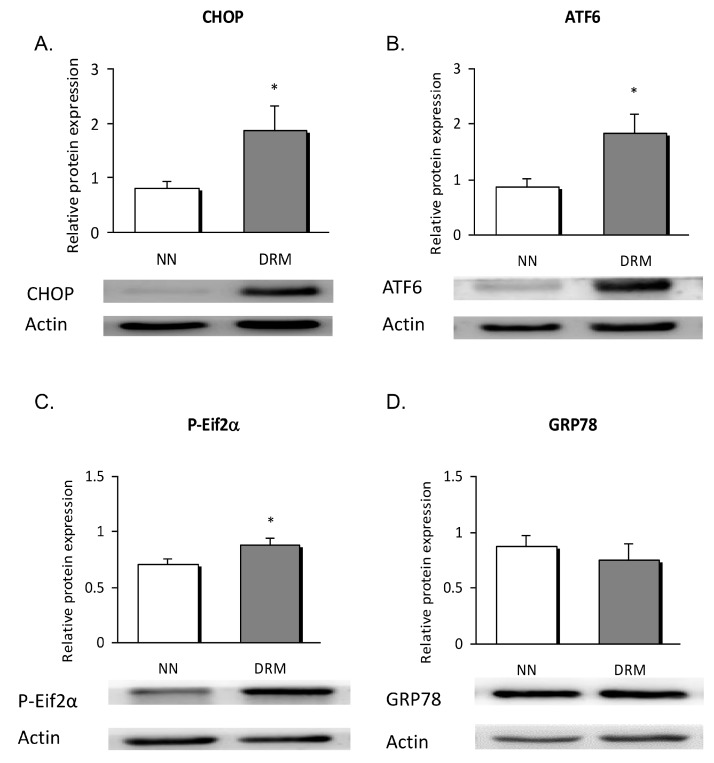
Leukocyte expression of ER stress proteins in NN and DRM subjects. (**A**) CHOP protein expression relative to actin protein expression, in NN vs DRM patients. (**B**) ATF6 protein expression relative to actin protein expression in NN vs DRM patients. (**C**) P-Eif2α protein expression relative to actin protein expression in NN vs DRM patients. (**D**) GRP78 protein expression relative to actin protein expression in NN vs DRM patients. * *p* < 0.05 vs. NN group using an unpaired *t*-test NN: Normonutrition; DRM: Disease-related malnutrition; ER: endoplasmic reticulum

To clarify whether metabolic disorders influence ER stress in leukocytes, protein and mRNA expression of UPR markers were determined. The protein expression data showed a significant rise in the signal of CHOP (*p* = 0.041), ATF6 (*p* = 0.038), and P-eIF2α (*p* = 0.030) in DRM subjects. No significant changes were detected regarding the GRP78 signal (Figure 3).

**Figure 4:**
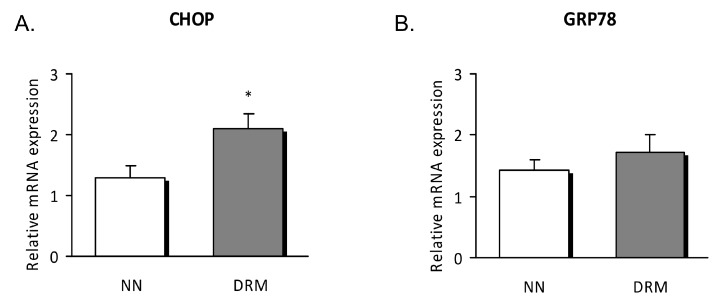
Relative mRNA expression in leukocytes of NN and DRM subjects. (**A**) CHOP gene expression relative to GAPDH gene expression in NN vs DRM patients. (**B**) GRP78 gene expression relative to GAPDH gene expression in NN vs DRM patients. * *p* < 0.05 vs. NN group using an unpaired *t*-test. NN: Normonutrition; DRM: Disease-related malnutrition.

mRNA expression revealed that CHOP was enhanced in malnourished patients compared to control subjects (*p* = 0.036), with no differences detected for the other genes analyzed (Figure 4). 

### 3.5. Correlation Analysis

In light of the abovementioned results, we explored a possible relationship among ER stress, mitochondrial function, inflammation, and biochemical and anthropometric parameters (Table 2). We found that CHOP levels were correlated with weight loss (*r* = 0.504, *p* = 0.012) inversely correlated with mid-upper arm circumference (*r* = −0.495, *p* = 0.014).

Remarkably, ATF6 protein levels were correlated with weight loss (*r* = 0.466, *p* = 0.025) and LDLc (*r* = 0.411, *p* = 0.046), as were P-eIF2α protein levels (*r* = 0.567, *p* = 0.005).

Percentage of weight loss was correlated with TNFα (*r* = 0.523, *p* < 0.001), IL6 (*r* = 0.443, *p* = 0.001), total ROS content (*r* = 0.505, *p* = 0.001), mitochondrial ROS content (*r* = 0.333, *p* = 0.047), Calcium content (*r* = 0.776, *p* < 0.001), TMRM (*r* = −0.471, *p* = 0.034), and oxygen consumption (*r* = −0.418, *p* = 0.001).

Transferrin correlated negatively with CHOP levels (*r* = −0.465, *p* = 0.017) and P-eIF2α levels (*r* = −0.383, *p* = 0.04). hsCRP was positively correlated with IL6 (*r* = 0.308, *p* = 0.031).

In short, all these correlations support a relationship between weight loss and with both increased inflammation and an undermined function of mitochondria and the ER.

## 4. Discussion

This study provides evidence that DRM is characterized by marked weight loss and lower levels of albumin and transferrin compared to values in a normonourished outpatient population. Moreover, in the leukocytes of DRM patients, markers of inflammation are exacerbated and express markers of undermined mitochondrial function and ER stress.

The alteration of nutritional status induces anthropometric and biochemical changes that need to be interpreted to make a correct assessment and diagnosis of DRM [12]. As expected, we observed significant weight loss and decreases in tricipital skin thickness, mid-upper arm circumference, arm muscular circumference, and calf circumference in malnourished patients comparing to those with normal nutritional status. Regarding biochemical markers of protein malnutrition, there was a slight decrease in albumin and transferrin in the serum of malnourished subjects, which is in line with previous reports [13,14,15,16,17]. These biomarkers are not diagnostic according to ASPEN criteria if the population presents caloric malnutrition (80.2% of our DRM population) and this is why we have not classified our patients according to the levels of these markers. But they give us information about hepatic metabolism and reinforce the malnurtured state of these subjects.

Growing evidence associates DRM with an underlying inflammatory state whose severity and persistence ends in a reduction of BMI, associated, in turn, with functional alterations [18,19,20,21]. This type of malnutrition is, in part, explained by a decrease in food intake, and is closely linked to the influence of inflammation on the metabolism of nutrients. In this sense, inflammation contributes to further protein loss due to the increased catabolism and hyporexia that it generates, thus accelerating the malnutrition process [22]. In this work, we have observed significant differences between DRM and NN subjects in hsCRP, as well as in the pro-inflammatory cytokines IL6 and TNFα and in the cell adhesion molecules ICAM-1 and VCAM-1. Recently, Epifanio et al. (2018) [21] confirmed that inflammation, when assessed by hsCRP, is not only related to cardiometabolic alterations but is also one of the key stages in the development of malnutrition in haemodialysis. Moreover, they established that oxidative stress contributes to enhanced inflammation, as they observed increased levels of serum gamma-glutamyl transferase and malondialdehyde [21]. Thus, the presence of this underlying inflammatory state could restrict the effectiveness of nutritional interventions and, together with malnutrition, may undermine medical treatment and patient recovery, as suggested by other reports [3,23].

On the other hand, in the presence of DRM, neurological, endocrine, inflammatory, and immunological components act synergistically in the metabolic response to stress. Immune cells, especially leukocytes, release pro-inflammatory cytokines (TNFα, IL-1, and IL-6) and ROS, generating inflammation, increased oxidative stress, ER stress, and cellular apoptosis. As reported by our results, an increase in ROS in malnourished patients has also been described, and independent pathways link it to the malnutrition disease [24]. The state of chronic inflammation activates the immune system, which leads to a slight increase in ROS production. This state, together with an abrupt reduction in the intake of carbohydrates, proteins, and vitamins, and the subsequent decrease of antioxidant mechanisms, could constitute some of the mechanisms underlying the increase in ROS. Moreover, multiple micronutrient deficiency or redistribution has been reported to occur in critically ill children displaying high levels of oxidative stress, in which there is a significant trend toward a decrease in plasma levels of micronutrients in parallel with an increase in the intensity of oxidative stress [25]. Our data of mitochondrial membrane potential, ROS, and oxygen consumption also confirm an undermined mitochondrial function in DRM subjects. At this point, it is important to note that a rise in both total and mitochondrial ROS and mitochondrial dysfunction has been related to the appearance of numerous diseases such as arteriosclerosis, diabetes mellitus, and cancer [26], which suggests that these molecules can be both a cause and consequence of disease. Given the importance of the mitochondrial function, it is necessary to explore this issue deeper to determine the importance of mitochondria integrity, including functional studies using inhibitors and activators of the respiratory chain.

ER stress is currently considered an important mediator of many pathophysiological conditions, including metabolic diseases. CHOP is a link between cytokine-induced inflammation and ER stress [10,27]. In line with this, our results show a remarkable rise in the protein expression of CHOP, ATF6, and the phosphorylated form of eIF2α in subjects with vs. those without DRM. Somewhat unexpectedly, we did not observe significant changes in GRP78, an important mediator of the UPR response that increases ER folding capacity. However, we must bear in mind that the pathologic background of our DRM patients could have promoted apoptotic and inflammatory pathways, rather than restoration of cellular homeostasis. Calcium content was significantly enhanced in the DRM group, thus suggesting that ER function has been compromised. Nevertheless, we did not determine the source of calcium overload, and so we intend to explore this aspect in future studies by employing specific probes. Interestingly, we found weight loss, mid-upper arm circumference, and transferrin to be significantly correlated with inflammatory and ER markers. These results highlight a relationship of anthropometrical and biochemical measures with biological markers involved in cellular function.

The present study presents several limitations. The reduced number of our study population, basically due to the low prevalence of malnourishment in our outpatient population [15], limits the scope of this work. Moreover, the population suffered a diverse array of chronic diseases, making it difficult to compare this study with similar ones. It would be of great interest to determine whether or not malnourishment, rather than the disease itself, was the cause of the changes we observed. Additionally, a more detailed analysis of mitochondrial function and membrane potential rescue by an antioxidant will be addressed in future studies.

## 5. Conclusions

In summary, the findings of this work expand the existing knowledge of the cellular processes at work in the leukocytes of DRM subjects. They provide evidence that inflammation and oxidative stress increase together with the induction of ER stress, underlining the importance of nutritional assessment and anti-inflammatory therapies to ameliorate these effects in subjects with DRM.

## Figures and Tables

**Table 1:** Distribution of the different diseases presented by NN and DRM groups.

	NN	DRM
Neurological disease	18.80%	16.30%
Pneumological disease	31.00%	20.90%
Digestive disease	23.10%	20.90%
Endocrine disease	5.10%	7.00%
Cardiovascular disease	10.70%	7.00%
Viral infection	6.80%	12.80%
Haematologic disease	0.50%	1.20%
Neoplasms	1.50%	11.60%
Other	2.50%	2.30%

Data are expressed as percentage (%) of the total subjects from each group. DRM: Disease-related malnutrition; NN: Normonutrition.

**Table 2:** Comparison of anthropometrical, biochemical, and metabolic parameters between normonourished and disease-related malnutrition subjects.

	NN (*n* = 37)	DRM (*n* = 45)	*p*-Value
Age (years)	57.9 ± 19.4	65.7 ± 15.9	0.15
Weight (kg)	65.4 ± 14	55.6 ± 10.6	0.021
Weight loss (%)	2.9 ± 3	26.2 ± 5.5	<0.001
BMI (kg/m^2^)	25.9 ± 16.3	21.2 ± 2.8	<0.001
Triceps skin thickness (mm)	19.3 ± 6.9	13.6 ± 4.9	<0.001
Mid-upper arm circumference (cm)	28.8 ± 4.7	24.7 ± 2.8	<0.001
Arm muscular circumference (cm)	22.8 ± 3.4	20.2 ± 3.1	<0.001
Calf circumference (cm)	36.3 ± 4.1	31 ± 2.86	<0.001
Albumin (g/dL)	4.2 ± 0.3	3.9 ± 0.6	<0.001
Prealbumin (mg/dL)	24.2 ± 5	23.3 ± 6.7	0.470
Transferrin (mg/dL)	267.3 ± 64.8	236 ± 63.6	0.031
Total cholesterol (mg/dL)	185.9 ± 32.2	184.8 ± 43.9	0.865
LDLc (mg/dL)	110.5 ± 29.1	112.3 ± 35.8	0.805
HDLc (mg/dL)	50.8 ± 14.9	50.3 ± 15.9	0.885
Triglycerides (mg/dL)	98.0 (83.8;126.0)	92.5(66.3;143.3)	0.330
hsCRP (mg/L)	2.8 ± 2.8	4.4 ± 3.5	0.038
Lymphocytes (10^9^/L)	2.1 ± 0.9	2.0 ± 0.8	0.710

Data are expressed as mean ± SD or median, 25th and 75th percentiles for parametric and non-parametric data, respectively. An unpaired *t*-test was employed to compare groups. *p*-value when comparing NN vs. DRM. DRM: Disease-related malnutrition; NN: Normonutrition; LDLc: Low density lipoprotein cholesterol; HDLc: High density lipoprotein cholesterol; hsCRP: high-sensitivity C-reactive protein.

## Abbreviations

AMP	arm muscle perimeter
ASPEN	American Society of Parenteral and Enteral Nutrition
ATF6	activating transcription factor 6
CHOP	CCAAT/enhancer binding protein (C/EBP) homologous protein
DCFH-DA	2′,7′-dichlorodihydrofluorescein diacetate
DRM	disease-related malnutrition
ER	endoplasmic reticulum
GRP78	78-KDa glucose-regulated protein
hsCRP	high-sensitivity C-reactive protein
ICAM-1	intercellular adhesion molecule-1
IL6	interleukin 6
MUAC	mid-upper arm circumference
NN	normonutrition
P-eIF2α	phosphorylated eukaryotic translation initiation factor 2 subunit 1 alpha
ROS	reactive oxygen species
TMRM	tetramethylrhodamine
TNFα	tumor necrosis factor α
TST	triceps skinfold thickness
UPR	unfolded protein response
VCAM-1	vascular cell adhesion molecule-1
